# Supplementary figures and images for: Paramyxoviruses in Old World fruit bats (Pteropodidae): An open database and synthesis of sampling effort, viral positivity, and coevolution
Source: PLoS Negl Trop Dis. 2025 Nov 7;19(11):e0013698. doi: 10.1371/journal.pntd.0013698 (PMC12617870; doi:10.1371/journal.pntd.0013698)

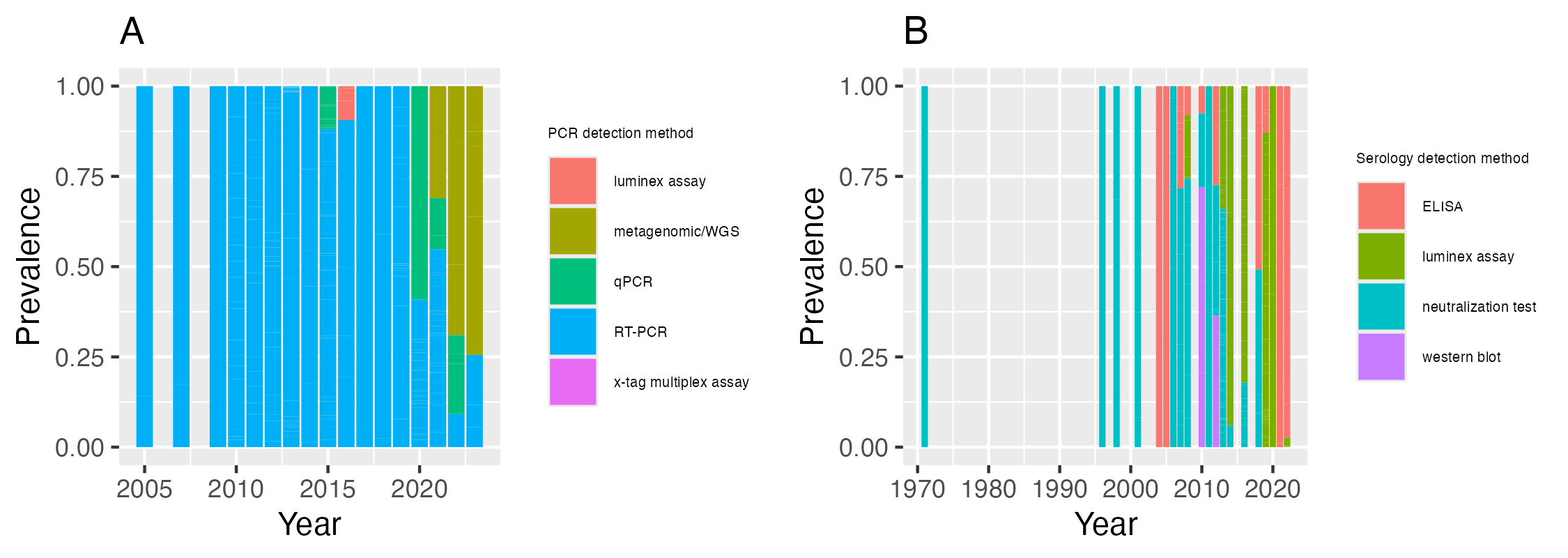

Supplement: S2 Fig — (TIFF) [file pntd.0013698.s002.tiff]
