# Supplementary material for: Paramyxoviruses in Old World fruit bats (Pteropodidae): An open database and synthesis of sampling effort, viral positivity, and coevolution
Source: PLoS Negl Trop Dis. 2025 Nov 7;19(11):e0013698. doi: 10.1371/journal.pntd.0013698 (PMC12617870; doi:10.1371/journal.pntd.0013698)
Supplement: S1 Text — (PDF) [file pntd.0013698.s008.pdf]

Reference list for “pteroparamyxo” dataset (Pteropodidae–paramyxovirus interactions)

Amman, B. R., C. G. Albariño, B. H. Bird, et al. 2015. “A Recently Discovered Pathogenic Paramyxovirus, Sosuga Virus, Is Present in Rousettus Aegyptiacus Fruit Bats at Multiple Locations in Uganda.” Journal of Wildlife Diseases 51 (3): 774–79. https://doi.org/10.7589/2015-02-044.

Anderson, D. E., A. Islam, G. Crameri, et al. 2019. “Isolation and Full-Genome Characterization of Nipah Viruses from Bats, Bangladesh.” Emerging Infectious Diseases 25 (1): 166–170. https://doi.org/10.3201/eid2501.180267.

Anthony, S. J., J. H. Epstein, K. A. Murray, et al. 2013. “A Strategy To Estimate Unknown Viral Diversity in Mammals.” mBio 4 (5): 10.1128/mbio.00598-13. https://doi.org/10.1128/mbio.00598-13.

Baker, K. S., S. Todd, G. Marsh, et al. 2012. “Co-Circulation of Diverse Paramyxoviruses in an Urban African Fruit Bat Population.” Journal of General Virology 93 (4): 850–56. https://doi.org/10.1099/vir.0.039339-0.

Baker, K. S., R. Suu-Ire, J. Barr, et al. 2014. “Viral Antibody Dynamics in a Chiropteran Host.” Journal of Animal Ecology 83 (2): 415–28. https://doi.org/10.1111/1365-2656.12153.

Baker, K. S., M. Tachedjian, J. Barr, et al. 2020. “Achimota Pararubulavirus 3: A New Bat-Derived Paramyxovirus of the Genus Pararubulavirus.” Viruses 12 (11): 11. https://doi.org/10.3390/v12111236.

Baker, K. S., S. Todd, G. A. Marsh, et al. 2013. “Novel, Potentially Zoonotic Paramyxoviruses from the African Straw-Colored Fruit Bat Eidolon Helvum.” Journal of Virology 87 (3): 1348–58. https://doi.org/10.1128/jvi.01202-12.

Barr, J. A., C. Smith, G. A. Marsh, H. Field, and L. F. Wang. 2012. “Evidence of Bat Origin for Menangle Virus, a Zoonotic Paramyxovirus First Isolated from Diseased Pigs.” Journal of General Virology 93 (12): 2590–94. https://doi.org/10.1099/vir.0.045385-0.

Barr, J., C. Smith, I. Smith, et al. 2015. “Isolation of Multiple Novel Paramyxoviruses from Pteropid Bat Urine.” Journal of General Virology 96 (1): 24–29. https://doi.org/10.1099/vir.0.068106-0.

Boardman, W. S. J., M. L. Baker, V. Boyd, et al. 2020. “Seroprevalence of Three Paramyxoviruses; Hendra Virus, Tioman Virus, Cedar Virus and a Rhabdovirus, Australian Bat Lyssavirus, in a Range Expanding Fruit Bat, the Grey-Headed Flying Fox (Pteropus Poliocephalus).” PLOS ONE 15 (5): e0232339. https://doi.org/10.1371/journal.pone.0232339.

Breed, A. C., M. F. Breed, J. Meers, and H. E. Field. 2011. “Evidence of Endemic Hendra Virus Infection in Flying-Foxes (Pteropus Conspicillatus)—Implications for Disease Risk Management.” PLOS ONE 6 (12): e28816. https://doi.org/10.1371/journal.pone.0028816.

Breed, A. C., J. Meers, I. Sendow, et al. 2013. “The Distribution of Henipaviruses in Southeast Asia and Australasia: Is Wallace’s Line a Barrier to Nipah Virus?” PLOS ONE 8 (4): e61316. https://doi.org/10.1371/journal.pone.0061316.

Breed, A. C., M. Yu, J. A. Barr, G. Crameri, C. M. Thalmann, and L. F. Wang. 2010. “Prevalence of Henipavirus and Rubulavirus Antibodies in Pteropid Bats, Papua New Guinea.” Emerging Infectious Diseases 16 (12): 1997-1999. https://doi.org/10.3201/eid1612.100879.

Brook, C. E., H. C. Ranaivoson, C. C. Broder, et al. 2019. “Disentangling Serology to Elucidate Henipa- and Filovirus Transmission in Madagascar Fruit Bats.” Journal of Animal Ecology 88 (7): 1001–16. https://doi.org/10.1111/1365-2656.12985.

Burroughs, A. L., P. A. Durr, V. Boyd, et al. 2016. “Hendra Virus Infection Dynamics in the Grey-Headed Flying Fox (Pteropus Poliocephalus) at the Southern-Most Extent of Its Range: Further Evidence This Species Does Not Readily Transmit the Virus to Horses.” PLOS ONE 11 (6): e0155252. https://doi.org/10.1371/journal.pone.0155252.

Cappelle, J., T. Hoem, V. Hul, et al. 2020. “Nipah Virus Circulation at Human–Bat Interfaces, Cambodia.” Bulletin of the World Health Organization 98 (8): 539–47. https://doi.org/10.2471/BLT.20.254227.

Chua, K. B. 2003. “A Novel Approach for Collecting Samples from Fruit Bats for Isolation of Infectious Agents.” Microbes and Infection 5 (6): 487–90. https://doi.org/10.1016/S1286-4579(03)00067-4.

Chua, K. B., C. L. Koh, P. S. Hooi, et al. 2002. “Isolation of Nipah Virus from Malaysian Island Flying-Foxes.” Microbes and Infection 4 (2): 145–51. https://doi.org/10.1016/S1286-4579(01)01522-2.

Chua, K. B., L. F. Wang, S. K. Lam, et al. 2001. “Tioman Virus, a Novel Paramyxovirus Isolated from Fruit Bats in Malaysia.” Virology 283 (2): 215–29. https://doi.org/10.1006/viro.2000.0882.

Conrardy, C., Y. Tao, I. V. Kuzmin, et al. 2014. “Molecular Detection of Adenoviruses, Rhabdoviruses, and Paramyxoviruses in Bats from Kenya.” The American Journal of Tropical Medicine and Hygiene 91 (2): 258–266. https://doi.org/10.4269/ajtmh.13-0664.

Cui, X., K. Fan, X. Liang, et al. 2023. “Virus Diversity, Wildlife-Domestic Animal Circulation and Potential Zoonotic Viruses of Small Mammals, Pangolins and Zoo Animals.” Nature Communications 14 (1): 2488. https://doi.org/10.1038/s41467-023-38202-4.

Drexler, J. F., V. M. Corman, F. Gloza-Rausch, et al. 2009. “Henipavirus RNA in African Bats.” PLOS ONE 4 (7): e6367. https://doi.org/10.1371/journal.pone.0006367.

Drexler, J. F., V. M. Corman, M. A. Müller, et al. 2012. “Bats Host Major Mammalian Paramyxoviruses.” Nature Communications 3 (1): 796. https://doi.org/10.1038/ncomms1796.

Edson, D., A. J. Peel, L. Huth, et al. 2019. “Time of Year, Age Class and Body Condition Predict Hendra Virus Infection in Australian Black Flying Foxes (Pteropus Alecto).” Epidemiology & Infection 147: e240. https://doi.org/10.1017/S0950268819001237.

Edson, D., H. Field, L. McMichael, D. Jordan, et al. 2015. “Flying-Fox Roost Disturbance and Hendra Virus Spillover Risk.” PLOS ONE 10 (5): e0125881. https://doi.org/10.1371/journal.pone.0125881.

Edson, D., H. Field, L. McMichael, M. Vidgen, et al. 2015. “Routes of Hendra Virus Excretion in Naturally-Infected Flying-Foxes: Implications for Viral Transmission and Spillover Risk.” PLOS ONE 10 (10): e0140670. https://doi.org/10.1371/journal.pone.0140670.

Epstein, J. H., S. J. Anthony, A. Islam, et al. 2020. “Nipah Virus Dynamics in Bats and Implications for Spillover to Humans.” Proceedings of the National Academy of Sciences 117 (46): 29190–201. https://doi.org/10.1073/pnas.2000429117.

Epstein, J. H., M. L. Baker, C. Zambrana-Torrelio, et al. 2013. “Duration of Maternal Antibodies against Canine Distemper Virus and Hendra Virus in Pteropid Bats.” PLOS ONE 8 (6): e67584. https://doi.org/10.1371/journal.pone.0067584.

Epstein, J. H., V. Prakash, C. S. Smith, et al. 2008. “Henipavirus Infection in Fruit Bats (Pteropus Giganteus), India.” Emerging Infectious Diseases 14 (8): 1309–1311. https://doi.org/10.3201/eid1408.071492.

Field, H., C. de Jong, D. Melville, et al. 2011. “Hendra Virus Infection Dynamics in Australian Fruit Bats.” PLOS ONE 6 (12): e28678. https://doi.org/10.1371/journal.pone.0028678.

Field, H., C. E. de Jong, K. Halpin, and C. S. Smith. 2013. “Henipaviruses and Fruit Bats, Papua New Guinea.” Emerging Infectious Diseases 19 (4): 670–71. https://doi.org/10.3201/eid1904.111912.

Field, H., D. Jordan, D. Edson, et al. 2015. “Spatiotemporal Aspects of Hendra Virus Infection in Pteropid Bats (Flying-Foxes) in Eastern Australia.” PLOS ONE 10 (12): e0144055. https://doi.org/10.1371/journal.pone.0144055.

Foo, R., Y. Y. Hey, J. H. J. Ng, et al. 2022. “Establishment of a Captive Cave Nectar Bat (Eonycteris Spelaea) Breeding Colony in Singapore.” Journal of the American Association for

Laboratory Animal Science: JAALAS 61 (4): 344–52. https://doi.org/10.30802/AALAS-JAALAS-21-000090.

Gibson, L., M. P. Ribas, J. Kemp, et al. 2021. “Persistence of Multiple Paramyxoviruses in a Closed Captive Colony of Fruit Bats (Eidolon Helvum).” Viruses 13 (8): 8. https://doi.org/10.3390/v13081659.

Glennon, Emma E., Daniel J. Becker, Alison J. Peel, et al. 2019. “What Is Stirring in the Reservoir? Modelling Mechanisms of Henipavirus Circulation in Fruit Bat Hosts.” Philosophical Transactions of the Royal Society B: Biological Sciences 374 (1782): 20190021. https://doi.org/10.1098/rstb.2019.0021.

Gokhale, M., M. Sreelekshmy, A. B. Sudeep, et al. 2021. “Detection of Possible Nipah Virus Infection in Rousettus Leschenaultii and Pipistrellus Pipistrellus Bats in Maharashtra, India.” Journal of Infection and Public Health 14 (8): 1010–12. https://doi.org/10.1016/j.jiph.2021.05.001.

Gokhale, M., A. B. Sudeep, B. Mathapati, et al. 2022. “Serosurvey for Nipah Virus in Bat Population of Southern Part of India.” Comparative Immunology, Microbiology and Infectious Diseases 85: 101800. https://doi.org/10.1016/j.cimid.2022.101800.

Goldspink, L. K., D. W. Edson, M. E. Vidgen, J. Bingham, H. E. Field, and C. S. Smith. 2015. “Natural Hendra Virus Infection in Flying-Foxes - Tissue Tropism and Risk Factors.” PLOS ONE 10 (6): e0128835. https://doi.org/10.1371/journal.pone.0128835.

Halpin, K., P. L. Young, H. E. Field, and J. S. Mackenzie. 2000. “Isolation of Hendra Virus from Pteropid Bats: A Natural Reservoir of Hendra Virus.” Journal of General Virology 81 (8): 1927–32. https://doi.org/10.1099/0022-1317-81-8-1927.

Hasebe, F., N. T. T. Thuy, S. Inoue, et al. 2012. “Serologic Evidence of Nipah Virus Infection in Bats, Vietnam.” Emerging Infectious Diseases 18 (3): 536–537. https://doi.org/10.3201/eid1803.111121.

Hayman, D. T. S., R. Suu-Ire, A. C. Breed, et al. 2008. “Evidence of Henipavirus Infection in West African Fruit Bats.” PLOS ONE 3 (7): e2739. https://doi.org/10.1371/journal.pone.0002739.

Hoarau, A. O. G., S. M. Goodman, D. Al Halabi, et al. 2021. “Investigation of Astrovirus, Coronavirus and Paramyxovirus Co-Infections in Bats in the Western Indian Ocean.” Virology Journal 18 (1): 205. https://doi.org/10.1186/s12985-021-01673-2.

Homaira, N., M. Rahman, M. J. Hossain, et al. 2010. “Nipah Virus Outbreak with Person-to-Person Transmission in a District of Bangladesh, 2007.” Epidemiology & Infection 138 (11): 1630–36. https://doi.org/10.1017/S0950268810000695.

Hsu, V. P., M. J. Hossain, U. D. Parashar, et al. 2004. “Nipah Virus Encephalitis Reemergence, Bangladesh.” Emerging Infectious Diseases 10 (12): 2082–2087. https://doi.org/10.3201/eid1012.040701.

Iehlé, C., G. Razafitrimo, J Razainirina, et al. 2007. “Henipavirus and Tioman Virus Antibodies in Pteropodid Bats, Madagascar.” Emerging Infectious Diseases 13 (1): 159. https://doi.org/10.3201/eid1301.060791.

Johnson, R. I., M. Tachedjian, B. Rowe, et al. 2018. “Alston Virus, a Novel Paramyxovirus Isolated from Bats Causes Upper Respiratory Tract Infection in Experimentally Challenged Ferrets.” Viruses 10 (12): 12. https://doi.org/10.3390/v10120675.

Jolma, E. R., L. Gibson, R. D. Suu-Ire, et al. 2021. “Longitudinal Secretion of Paramyxovirus RNA in the Urine of Straw-Coloured Fruit Bats (Eidolon Helvum).” Viruses 13 (8): 8. https://doi.org/10.3390/v13081654.

Kashiwazaki, Y., Y. N. Na, N. Tanimura, and T. Imada. 2004. “A Solid-Phase Blocking ELISA for Detection of Antibodies to Nipah Virus.” Journal of Virological Methods 121 (2): 259–61. https://doi.org/10.1016/j.jviromet.2004.06.015.

Kia, G. S. N., Y. Tao, J. U. Umoh, J. K. P. Kwaga, and S. Tong. 2021. “Identification of Coronaviruses, Paramyxoviruses, Reoviruses, and Rotaviruses among Bats in Nigeria.” The American Journal of Tropical Medicine and Hygiene 104 (3): 1106–1110. https://doi.org/10.4269/ajtmh.19-0872.

Kirkland, P. D., R. J. Love, A. W. Philbey, A. D. Ross, R. J. Davis, and K. G. Hart. 2001. “Epidemiology and Control of Menangle Virus in Pigs.” Australian Veterinary Journal 79 (3): 199–206. https://doi.org/10.1111/j.1751-0813.2001.tb14580.x.

Latinne, A., N. T. T. Nga, N. V. Long, et al. 2023. “One Health Surveillance Highlights Circulation of Viruses with Zoonotic Potential in Bats, Pigs, and Humans in Viet Nam.” Viruses 15 (3): 3. https://doi.org/10.3390/v15030790.

Lau, S. K. P., P. C. Y. Woo, B. H. L. Wong, et al. 2010. “Identification and Complete Genome Analysis of Three Novel Paramyxoviruses, Tuhoko Virus 1, 2 and 3, in Fruit Bats from China.” Virology 404 (1): 106–16. https://doi.org/10.1016/j.virol.2010.03.049.

Li, Y., J. Wang, A. C. Hickey, et al. 2008. “Antibodies to Nipah or Nipah-like Viruses in Bats, China.” Emerging Infectious Diseases 14 (12): 1974–76. https://doi.org/10.3201/eid1412.080359.

Madera, S., A. Kistler, H. C. Ranaivoson, et al. 2022. "Discovery and Genomic Characterization of a Novel Henipavirus, Angavokely Virus, from Fruit Bats in Madagascar." Journal of Virology 96 (18): e00921-22. https://doi.org/10.1128/jvi.00921-22.

Markotter, W., M. Geldenhuys, P. J. van Vuren, et al. 2019. "Paramyxo- and Coronaviruses in Rwandan Bats." Tropical Medicine and Infectious Disease 4 (3): 3. https://doi.org/10.3390/tropicalmed4030099.

Marsh, G. A., C. de Jong, J. A. Barr, et al. 2012. "Cedar Virus: A Novel Henipavirus Isolated from Australian Bats." PLOS Pathogens 8 (8): e1002836. https://doi.org/10.1371/journal.ppat.1002836.

McCutchan, J. L., M. A. Knox, Alivereti Naikatini, David T. S. Hayman, and Brett D. Gartrell. 2023. "Molecular Evidence of Leptospira Spp. in Isolated Fijian Bats." Journal of Wildlife Diseases 59 (1): 202–6. https://doi.org/10.7589/JWD-D-22-00038.

McEvoy, J. F., J. C. Kishbaugh, M. T. Valitutto, et al. 2021. "Movements of Indian Flying Fox in Myanmar as a Guide to Human-Bat Interface Sites." EcoHealth 18 (2): 204–16. https://doi.org/10.1007/s10393-021-01544-w.

McKee, C. D., A. Islam, M. Z. Rahman, et al. 2022. "Nipah Virus Detection at Bat Roosts after Spillover Events, Bangladesh, 2012–2019." Emerging Infectious Diseases 28 (7): 1384–1392. https://doi.org/10.3201/eid2807.212614.

McMichael, L., D. Edson, D. Mayer, et al. 2017. "Physiologic Biomarkers and Hendra Virus Infection in Australian Black Flying Foxes (Pteropus Alecto)." Journal of Wildlife Diseases 53 (1): 111–20. https://doi.org/10.7589/2016-05-100.

McMichael, L., D. Edson, C. Smith, et al. 2017. "Physiological Stress and Hendra Virus in Flying-Foxes (Pteropus Spp.), Australia." PLOS ONE 12 (8): e0182171. https://doi.org/10.1371/journal.pone.0182171.

Mélade, J., N. Wieseke, B. Ramasindrazana, et al. 2016. "An Eco-Epidemiological Study of Morbilli-Related Paramyxovirus Infection in Madagascar Bats Reveals Host-Switching as the Dominant Macro-Evolutionary Mechanism." Scientific Reports 6 (1): 23752. https://doi.org/10.1038/srep23752.

Mendenhall, I. H., D. L. H. Wen, J. Jayakumar, et al. 2019. "Diversity and Evolution of Viral Pathogen Community in Cave Nectar Bats (Eonycteris Spelaea)." Viruses 11 (3): 3. https://doi.org/10.3390/v11030250.

Mishra, N., S. F. Fagbo, A. N. Alagaili, et al. 2019. "A Viral Metagenomic Survey Identifies Known and Novel Mammalian Viruses in Bats from Saudi Arabia." PLOS ONE 14 (4): e0214227. https://doi.org/10.1371/journal.pone.0214227.

Mortlock, M., M. Dietrich, J. Weyer, J. T. Paweska, and W. Markotter. 2019. “Co-Circulation and Excretion Dynamics of Diverse Rubula- and Related Viruses in Egyptian Rousette Bats from South Africa.” Viruses 11 (1): 1. https://doi.org/10.3390/v11010037.

Mortlock, M., M. Geldenhuys, M. Dietrich, et al. 2021. “Seasonal Shedding Patterns of Diverse Henipavirus-Related Paramyxoviruses in Egyptian Rousette Bats.” Scientific Reports 11 (1): 24262. https://doi.org/10.1038/s41598-021-03641-w.

Mortlock, M., I. V. Kuzmin, J. Weyer, et al. 2015. “Novel Paramyxoviruses in Bats from Sub-Saharan Africa, 2007–2012.” Emerging Infectious Diseases 21 (10): 1840–1843. https://doi.org/10.3201/eid2110.140368.

Mourya, D. T., P. Yadav, A. B. Sudeep, et al. 2019. “Spatial Association Between a Nipah Virus Outbreak in India and Nipah Virus Infection in Pteropus Bats.” Clinical Infectious Diseases 69 (2): 378–79. https://doi.org/10.1093/cid/ciy1093.

Muleya, W., M. Sasaki, Y. Orba, et al. 2014. “Molecular Epidemiology of Paramyxoviruses in Frugivorous Eidolon Helvum Bats in Zambia.” Journal of Veterinary Medical Science 76 (4): 611–14. https://doi.org/10.1292/jvms.13-0518.

Olival, K. J., A. Latinne, A. Islam, et al. 2020. “Population Genetics of Fruit Bat Reservoir Informs the Dynamics, Distribution and Diversity of Nipah Virus.” Molecular Ecology 29 (5): 970–85. https://doi.org/10.1111/mec.15288.

Olson, J. G., C. E. Rupprecht, P. E. Rollin, et al. 2002. “Antibodies to Nipah-Like Virus in Bats (Pteropus Lylei), Cambodia.” Emerging Infectious Diseases 8 (9): 987–988. https://doi.org/10.3201/eid0809.010515.

Paskey, A. C., X. F. Lim, J. H. J. Ng, et al. 2023. “Genomic Characterization of a Relative of Mumps Virus in Lesser Dawn Bats of Southeast Asia.” Viruses 15 (3): 3. https://doi.org/10.3390/v15030659.

Pavri, K. M., K. R. P. Singh, and F. B. Hollinger. 1971. Isolation of a New Parainfluenza Virus from a Frugivorous Bat, Rousettus Leschenaulti, Collected at Poona, India. The American Journal of Tropical Medicine and Hygiene 20 (1): 125–130. https://doi.org/10.4269/ajtmh.1971.20.125.

Peel, A. J., K. S. Baker, G. Crameri, et al. 2012. “Henipavirus Neutralising Antibodies in an Isolated Island Population of African Fruit Bats.” PLOS ONE 7 (1): e30346. https://doi.org/10.1371/journal.pone.0030346.

Peel, A. J., K. S. Baker, D. T. S. Hayman, et al. 2016. “Bat Trait, Genetic and Pathogen Data from Large-Scale Investigations of African Fruit Bats, Eidolon Helvum.” Scientific Data 3 (1): 160049. https://doi.org/10.1038/sdata.2016.49.

Peel, A. J., T. J. McKinley, K. S. Baker, et al. 2013. “Use of Cross-Reactive Serological Assays for Detecting Novel Pathogens in Wildlife: Assessing an Appropriate Cutoff for Henipavirus Assays in African Bats.” Journal of Virological Methods 193 (2): 295–303. https://doi.org/10.1016/j.jviromet.2013.06.030.

Peel, A. J., D. R. Sargan, K. S. Baker, et al. 2013. “Continent-Wide Panmixia of an African Fruit Bat Facilitates Transmission of Potentially Zoonotic Viruses.” Nature Communications 4 (1): 2770. https://doi.org/10.1038/ncomms3770.

Peel, A. J., K. Wells, J. Giles, et al. 2019. “Synchronous Shedding of Multiple Bat Paramyxoviruses Coincides with Peak Periods of Hendra Virus Spillover.” Emerging Microbes & Infections 8 (1): 1314–23. https://doi.org/10.1080/22221751.2019.1661217.

Peel, A. J., C. K. Yinda, E. J. Annand, et al. 2022. “Novel Hendra Virus Variant Circulating in Black Flying Foxes and Grey-Headed Flying Foxes, Australia.” Emerging Infectious Diseases 28 (5): 1043–1047. https://doi.org/10.3201/eid2805.212338.

Pernet, O., B. S. Schneider, S. M. Beaty, et al. 2014. “Evidence for Henipavirus Spillover into Human Populations in Africa.” Nature Communications 5 (1): 5342. https://doi.org/10.1038/ncomms6342.

Philbey, A. W., P. D. Kirkland, A. D. Ross, et al. 1998. “An Apparently New Virus (Family Paramyxoviridae) Infectious for Pigs, Humans, and Fruit Bats.” Emerging Infectious Diseases Journal 4 (2): 269–271. https://doi.org/10.3201/eid0402.980214.

Philbey, A. W., P. D. Kirkland, A. D. Ross, et al. 2008. “Infection with Menangle Virus in Flying Foxes (Pteropus Spp.) in Australia.” Australian Veterinary Journal 86 (11): 449–54. https://doi.org/10.1111/j.1751-0813.2008.00361.x.

Plowright, R. K, H. E Field, C. Smith, et al. 2008. “Reproduction and Nutritional Stress Are Risk Factors for Hendra Virus Infection in Little Red Flying Foxes (Pteropus Scapulatus).” Proceedings of the Royal Society B: Biological Sciences 275 (1636): 861–69. https://doi.org/10.1098/rspb.2007.1260.

Pulscher, L. A., A. J. Peel, K. Rose, et al. 2022. “Serological Evidence of a Pararubulavirus and a Betacoronavirus in the Geographically Isolated Christmas Island Flying-Fox (Pteropus Natalis).” Transboundary and Emerging Diseases 69 (5): e2366–77. https://doi.org/10.1111/tbed.14579.

Rahman, M. Z., M. M. Islam, M. E. Hossain, et al. 2021. “Genetic Diversity of Nipah Virus in Bangladesh.” International Journal of Infectious Diseases 102: 144–51. https://doi.org/10.1016/j.ijid.2020.10.041.

Rahman, S. A., L. Hassan, J. H. Epstein, et al. 2013. “Risk Factors for Nipah Virus Infection among Pteropid Bats, Peninsular Malaysia.” Emerging Infectious Diseases 19 (1): 51–60. https://doi.org/10.3201/eid1901.120221.

Rahman, S. A., S. S. Hassan, K. J. Olival, et al. 2010. “Characterization of Nipah Virus from Naturally Infected Pteropus Vampyrus Bats, Malaysia.” Emerging Infectious Diseases 16 (12): 1990–93. https://doi.org/10.3201/eid1612.091790.

Raut, C.G., P.D. Yadav, J.S. Towner, et al. 2012. “Isolation of a Novel Adenovirus from Rousettus Leschenaultii Bats from India.” Intervirology 55 (6): 488–90. https://doi.org/10.1159/000337026.

Reynes, J. M., D. Counor, S. Ong, et al. 2005. “Nipah Virus in Lyle’s Flying Foxes, Cambodia.” Emerging Infectious Diseases 11 (7): 1042–1047. https://doi.org/10.3201/eid1107.041350.

Sasaki, M., A. Setiyono, E. Handharyani, et al. 2012. “Molecular Detection of a Novel Paramyxovirus in Fruit Bats from Indonesia.” Virology Journal 9 (1): 240. https://doi.org/10.1186/1743-422X-9-240.

Sendow, I., H. E. Field, A. Adjid, et al. 2010. “Screening for Nipah Virus Infection in West Kalimantan Province, Indonesia.” Zoonoses and Public Health 57 (7–8): 499–503. https://doi.org/10.1111/j.1863-2378.2009.01252.x.

Sendow, I., H. E. Field, J. Curran, et al. 2006. “Henipavirus in Pteropus Vampyrus Bats, Indonesia.” Emerging Infectious Diseases 12 (4): 711–712. https://doi.org/10.3201/eid1204.051181.

Sendow, I., A. Ratnawati, T. Taylor, et al. 2013. “Nipah Virus in the Fruit Bat Pteropus Vampyrus in Sumatera, Indonesia.” PLOS ONE 8 (7): e69544. https://doi.org/10.1371/journal.pone.0069544.

Shirai, J., A. L. Sohayati, A. L. M. Ali, M. N. Suriani, T. Taniguchi, and S. H. Sharifah. 2007. “Nipah Virus Survey of Flying Foxes in Malaysia.” Japan Agricultural Research Quarterly: JARQ 41 (1): 69–78. https://doi.org/10.6090/jarq.41.69.

Smith, I., A. Broos, C. de Jong, et al. 2011. “Identifying Hendra Virus Diversity in Pteropid Bats.” PLOS ONE 6 (9): e25275. https://doi.org/10.1371/journal.pone.0025275.

Sohayati, A. R., L. Hassan, S. H. Sharifah, et al. 2011. “Evidence for Nipah Virus Recrudescence and Serological Patterns of Captive Pteropus Vampyrus.” Epidemiology and Infection 139 (10): 1570–79. https://doi.org/10.1017/S0950268811000550.

Su, H., Y. Wang, Y. Han, Q. Jin, F. Yang, and Z. Wu. 2023. “Discovery and Characterization of Novel Paramyxoviruses from Bat Samples in China.” Virologica Sinica 38 (2): 198–207. https://doi.org/10.1016/j.virs.2023.01.002.

Sudeep, A. B., P. D. Yadav, M. D. Gokhale, et al. 2021. “Detection of Nipah Virus in Pteropus Medius in 2019 Outbreak from Ernakulam District, Kerala, India.” BMC Infectious Diseases 21 (1): 162. https://doi.org/10.1186/s12879-021-05865-7.

Tsang, S. M., D. H. W. Low, S. Wiantoro, et al. 2021. “Detection of Tioman Virus in Pteropus Vampyrus Near Flores, Indonesia.” Viruses 13 (4): 4. https://doi.org/10.3390/v13040563.

Vidgen, M. E., C. de Jong, K. Rose, J. Hall, H. E. Field, and C. S. Smith. 2015. “Novel Paramyxoviruses in Australian Flying-Fox Populations Support Host–Virus Co-Evolution.” Journal of General Virology 96 (7): 1619–25. https://doi.org/10.1099/vir.0.000099.

Wacharapluesadee, S., K. Boongird, S. Wanghongsa, et al. 2010. “A Longitudinal Study of the Prevalence of Nipah Virus in Pteropus Lylei Bats in Thailand: Evidence for Seasonal Preference in Disease Transmission.” Vector-Borne and Zoonotic Diseases 10 (2): 183–90. https://doi.org/10.1089/vbz.2008.0105.

Wacharapluesadee, S., S. Ghai, P. Duengkae, et al. 2021. “Two Decades of One Health Surveillance of Nipah Virus in Thailand.” One Health Outlook 3 (1): 12. https://doi.org/10.1186/s42522-021-00044-9.

Wacharapluesadee, S., and T. Hemachudha. 2007. “Duplex Nested RT-PCR for Detection of Nipah Virus RNA from Urine Specimens of Bats.” Journal of Virological Methods 141 (1): 97–101. https://doi.org/10.1016/j.jviromet.2006.11.023.

Wacharapluesadee, S., B. Lumlertdacha, K. Boongird, et al. 2005. “Bat Nipah Virus, Thailand.” Emerging Infectious Diseases 11 (12): 1949–1951. https://doi.org/10.3201/eid1112.050613.

Wacharapluesadee, S., T. Ngamprasertwong, T. Kaewpom, et al. 2017. “Genetic Characterization of Nipah Virus from Thai Fruit Bats (Pteropus Lylei).” Asian Biomedicine 7 (6): 813–19.

Wacharapluesadee, S., P. Samseeneam, M. Phermpool, et al. 2016. “Molecular Characterization of Nipah Virus from Pteropus Hypomelanus in Southern Thailand.” Virology Journal 13 (1): 53. https://doi.org/10.1186/s12985-016-0510-x.

Wang, J., D. E. Anderson, K. Halpin, et al. 2021. “A New Hendra Virus Genotype Found in Australian Flying Foxes.” Virology Journal 18 (1): 197. https://doi.org/10.1186/s12985-021-01652-7.

Waruhiu, C., S. Ommeh, V. Obanda, et al. 2017. “Molecular Detection of Viruses in Kenyan Bats and Discovery Of Novel Astroviruses, Caliciviruses and Rotaviruses.” Virologica Sinica 32 (2): 101–14. https://doi.org/10.1007/s12250-016-3930-2.

Weiss, S., K. Nowak, J. Fahr, et al. 2012. “Henipavirus-Related Sequences in Fruit Bat Bushmeat, Republic of Congo.” Emerging Infectious Diseases 18 (9): 1535–1536. https://doi.org/10.3201/eid1809.111607.

Wilkinson, D. A., S. Temmam, C. Lebarbenchon, et al. 2012. “Identification of Novel Paramyxoviruses in Insectivorous Bats of the Southwest Indian Ocean.” Virus Research 170 (1): 159–63. https://doi.org/10.1016/j.virusres.2012.08.022.

Yadav, P. D., C. G. Raut, A. M. Shete, et al. 2012a. “Detection of Nipah Virus RNA in Fruit Bat (Pteropus Giganteus) from India.” The American Journal of Tropical Medicine and Hygiene 87 (3): 576–578. https://doi.org/10.4269/ajtmh.2012.11-0416.

Yadav, P. D., R. R. Sahay, A. Balakrishnan, et al. 2022. “Nipah Virus Outbreak in Kerala State, India Amidst of COVID-19 Pandemic.” Frontiers in Public Health 10 (February). https://doi.org/10.3389/fpubh.2022.818545.

Yadav, P. D., A. M. Shete, G. A. Kumar, et al. 2016. “Nipah Virus Sequences from Humans and Bats during Nipah Outbreak, Kerala, India, 2018.” Emerging Infectious Diseases 25 (5): 1003–1006. https://doi.org/10.3201/eid2505.181076.

Yadav, P. D., P. Sarkale, D. Patil, et al. 2016. “Isolation of Tioman Virus from Pteropus Giganteus Bat in North-East Region of India.” Infection, Genetics and Evolution 45: 224–29. https://doi.org/10.1016/j.meegid.2016.09.010.

Yadav, P. D., A. Sudeep, M. Gokhale, et al. 2018. “Circulation of Nipah Virus in Pteropus Giganteus Bats in Northeast Region of India, 2015.” Indian Journal of Medical Research 147 (3): 318–20. https://doi.org/10.4103/ijmr.IJMR_1488_16.

Yob, J. M., H. Field, A. M. Rashdi, et al. 2001. “Nipah Virus Infection in Bats (Order Chiroptera) in Peninsular Malaysia.” Emerging Infectious Diseases 7 (3): 439–41. https://doi.org/10.3201/eid0703.010312.

Young, P. L., K. Halpin, P. W. Selleck, et al. 1996. “Serologic Evidence for the Presence in Pteropus Bats of a Paramyxovirus Related to Equine Morbillivirus.” Emerging Infectious Diseases 2 (3): 239–240. https://doi.org/10.3201/eid0203.960315.

Yuan, L., M. Li, L. Li, et al. 2014. "Evidence for Retrovirus and Paramyxovirus Infection of Multiple Bat Species in China." Viruses 6 (5): 5. https://doi.org/10.3390/v6052138.
