# Supplementary material for: Paramyxoviruses in Old World fruit bats (Pteropodidae): An open database and synthesis of sampling effort, viral positivity, and coevolution
Source: PLoS Negl Trop Dis. 2025 Nov 7;19(11):e0013698. doi: 10.1371/journal.pntd.0013698 (PMC12617870; doi:10.1371/journal.pntd.0013698)
Supplement: Attachment — Submitted filename: Juman et al. response to reviewers.pdf [file pntd.0013698.s010.pdf]

August 13, 2025

Dear Drs. Kamhawi and Brindley,

I have uploaded a revised version of our manuscript, "Paramyxoviruses in Old World fruit bats (Pteropodidae): an open database and synthesis of sampling effort, viral positivity, and coevolution" (PNTD-D-25-01418) into the Editorial Manager portal, and it should be ready for your inspection. In revising this manuscript for *PLOS Neglected Tropical Diseases*, we paid close attention to the three reviewer comments originally received from *PLOS Pathogens*. This led us to update our figures and supplementary files, add some additional clarifications to the text, and upload our database to GitHub where it can be accessed and updated by other researchers.

Below, we included all of the editor and reviewer comments, followed by our responses to them (in **bold**). Cited line numbers correspond to the revised manuscript, which has also been reorganized following guidelines for *PLOS Neglected Tropical Diseases*.

Thank you again for your consideration. Please do not hesitate to contact me if you have any questions.

Sincerely,
Maya Juman

**Editor comments**

I thank the authors for submitting this comprehensive review of paramyxoviruses in Pteropodidae bats, comprising 119 studies and describing the findings from a meta-analyses of the available data. The reviewers had split in their views of the usefulness of such a database, but I am inclined to agree with reviewers 2 and 3 that this compilation of studies could be a useful reference point for those in the field. However, reviewer 3 in particular raises some points that would be worth addressing, in particular some errors in the year of isolation of Hendra and Nipah, and a suggestion that the database could be made electronic to allow easier access. I would also urge the authors to consider if even a simple online implementation that allowed updates could be possible, as this would mean the database could be kept up to date for future studies, making it even more useful to the field (this could perhaps be implemented even on Github with additions/changes submitted via PR from the original authors or community).

**We thank the original editor for this positive feedback. In addition to its inclusion as supplementary material with this manuscript, our dataset is now housed in a public GitHub repository (https://github.com/mayajuman/pteroparamyxo). This facilitates potential updates in the future via pull requests by authors of new studies as well as release of new versions of the database as new studies are integrated. This is now mentioned on lines 471-472:**

**"Storing the dataset in this dynamic repository facilitates future updates and additional versions that include more recent studies."**

In addition, reviewer 1 makes a relevant point about the differences of methods used across these many studies - it would be worth including a comment about how this may impact drawing them together and the conclusions drawn.

**We have edited the discussion to address this limitation (lines 548-551):**

**"Our study is also inherently limited by between-study variation at a finer level than what is captured in our dataset and in many of the original studies (e.g., sampling protocols, PCR primers, laboratory protocols, etc)."**

Finally, I would recommend converting the Supplementary tables S1-S4 to CSV or TSV files if possible, as this will allow computational processing more easily by others, if they are interested (though I also recognise this sacrifices somewhat immediate readability).

**We have converted our supplementary tables to an Excel file that is readable but also facilitates easy extraction into CSV files for processing.**

**Reviewers' comments:**

Part I - Summary

Reviewer #1: See below

Reviewer #2: In this systematic review Juman et al. gathered literature published between 1971 and 2003 focusing on the investigation of paramyxoviruses in bats of the Pteropodidae family. In total they retained 1,482 records of prevalence or seroprevalence coming from 119 studies.

Their methodology is clear and precise. The authors clearly explain inclusion and exclusion criteria, how they investigated the effect of each factor. For their analysis, they took into account important biases and challenges.

The results emerging from this meta analysis are really important as they clearly set up the path for future studies and research questions that remain to be answered.

I don't have minor or major points to bring up. This is a really great work and the paper is well written. Congratulations to the authors for such a great job.

**We thank Reviewer 2 for the positive feedback!**

Reviewer #3: This is an ambitious paper, but I have thoroughly enjoyed reviewing it. Noting that I am not a modeler, my assessment of the models is very superficial.

The authors bring together a large amount of bat paramyxovirus virology study data with 1482 records gathered from 119 published studies. The backbone of the work could be described as a large literature review with more than 14 relevant fields in a spreadsheet capturing the data which has been extracted from the studies. This results in an excellent collation of studies (1971-2023) that have sampled wild pteropodids bats, with both positive and negative findings. Although they note that it is a static database.

It is a great idea to pool all the publicly available data from previous studies to construct a database and begin to interrogate it. The authors start by focusing on the sampling and other biases that affect where we can find a paramyxovirus in bats. The database also provides a valuable resource which can be used by others to interrogate according to their interests.

Turning this into a live database would be very welcomed. Are there any plans to turn this into a live database rather than a static one?

**See response to Associate Editor above. Using GitHub for data deposition here allows for future updates via pull requests as well as versioning of the database through inclusion of new studies. See lines 471-472 for brief discussion of this point.**

The spreadsheet of data was included as supplementary data. The authors describe that the database, 'pteroparamyxo' is PRISMA-compliant. Is it possible to access the database online, or are readers limited to the csv file which is supplied as supplementary data? More information around access to the database would be helpful.

**In addition to accompanying the manuscript as a supplementary file, the database lives on GitHub in a public repository (https://github.com/mayajuman/pteroparamyxo). We have now clarified this in the manuscript as well (lines 467-470):**

**"We sought to aggregate all published records of pteropodid–paramyxovirus associations and prevalence into a PRISMA-compliant, open, static database (Supplementary Fig. 1), included here as a supplementary file and also available in a public GitHub repository (Supplementary Data 1; https://github.com/mayajuman/pteroparamyxo)."**

With so many studies captured (119), it was impossible to cross check the accuracy of all of the data. However, with the studies I am most familiar with, I was able to confirm that these were captured accurately.

Part II – Major Issues: Key Experiments Required for Acceptance

Reviewer #1: This paper describes a new database (from published sources) of paramyxoviruses in Old World Pteropodidae bats and looks for correlates of PCR prevalence etc.

Although it is impressive that the authors have generated such a comprehensive database from published sources, and what they have done looks well done, in my opinion this paper suffers from two major shortcomings:

1. It is a very niche database. To be frank, the number of people who will utilise this database is going to very small. Sure, it's great to have it, but we are talking about one virus family in one host family. Hence, I am not convinced that this is really going to be of interest to a general audience, such as the readership of PLoS Pathogens. A database of all the viruses found in Pteropodidae bats would be much more important and interesting.

**The manuscript has now been transferred to a journal with a more specific audience, *PLOS Neglected Tropical Diseases*. Paramyxoviruses include henipaviruses which cause high**

**disease burdens in the tropics and remain understudied relative to other viral families such as coronaviruses, filoviruses, lyssaviruses, and poxviruses (based on a PubMed search, 31/7/25). We therefore believe that this manuscript will be of interest to the readership of *PLOS NTDs*.**

**While we recognize that the scope of this paper is taxonomically constrained to one viral family and one host family, we disagree with the notion that this is of "niche" interest. Nipah virus, in particular, has been on the WHO's and CEPI's priority lists for vaccine development for several years. More broadly, the recent proliferation of work focused on paramyxoviruses in Pteropodidae (as demonstrated in our study; see Fig. 1) suggests that the research community would benefit from a synthesis of the existing literature to inform future directions. Focusing our study on one viral family has allowed us to perform more specific and in-depth analyses (e.g., cophylogenetic analysis, viral taxonomic sampling biases, etc) and extract more detailed data than could be captured if the database covered all viral families.**

2. The major results found are either well known or of no particular importance. In particular, it is blindingly obvious that there is an understanding of African bats and an oversampling of henipaviruses. You really don't need a database to tell you this: it is evident from an even a cursory look at the literature. Indeed, I am never convinced by papers where the main result is to tell is the gaps in sampling. This is not a result of direct biological importance. Ditto with the co-phylogenetic analysis (and it is "co-divergence" not "co-evolution"). The analysis presented on this is pretty trivial and it is already well-known that there is a lot of host-jumping among these viruses and hosts. To be blunt then, this paper really didn't tell me anything that I did not know already.

**We appreciate that, for experts in the field, some of our results are largely confirmatory. Still, we believe in the usefulness of thoroughly testing hypotheses about sampling gaps and prevalence patterns. Not only do these results provide useful background for researchers or students newly interested in pteropodid-paramyxovirus relationships, they also provide a foundation and a justification for future studies. We disagree with the Reviewer's statement that gaps in sampling are not worth reporting. By statistically evaluating the taxonomic and geographic coverage of existing studies and highlighting resulting biases, we hope to encourage more study of neglected species and regions within this system. Much of what we know about pteropodid-paramyxovirus associations is driven by a disproportionate focus on certain bat and viral species (e.g., Hendra and Nipah viruses in *Pteropus*), and therefore the sampling gaps do directly impact our knowledge of biological phenomena.**

**Further, while the reviewer notes that host-jumping in this system is "well-known", some studies have hypothesized the opposite—that paramyxoviruses and pteropodid bats have co-evolved and that certain viral strains cluster in specific host groups (Vidgen et al. 2015, Journal of General Virology; Liang et al. 2021, BMC Ecology and Evolution). Our study investigates this hypothesis and presents intriguing evidence to the contrary.**

3. A technical concern is that the paper summarises over so many different studies that have used so many different laboratory techniques. For example, it must be the case that different PCR primers were used in different studies and that factors like this must contribute to some of the heterogeneities observed. While I agree with the overall results - because they are largely obvious - I am not convinced that a one size fits all approach will work.

**We appreciate Reviewer 1's concern, though this is a limitation inherent to any large-scale synthesis or meta-analysis of multiple studies. This is why we calculate and report between- and within-study heterogeneity in our meta-analyses of viral prevalence and seroprevalence, as these estimates can help identify within-study factors such as PCR conditions that affect viral positivity. We have now also added a sentence addressing this limitation to our discussion (lines 548-551):**

**"Our study is also inherently limited by between-study variation at a finer level than what is captured in our dataset and in many of the original studies (e.g., sampling protocols, PCR primers, laboratory protocols, etc)."**

Reviewer #2: (No Response)

Reviewer #3: I only found two errors in the manuscript, and they were both in Figure 1. A red line is placed on both graphs, and this represents when Hendra virus was first isolated from a bat. The date should be 1996 (not 2000). Likewise there is a blue line which represents when Nipah virus was first isolated from a bat. The date should be 2000 (not 2002). The dates in the supplementary data spreadsheet are correct. I think the author has used the year of publication instead of the year of isolation for these two lines.

**Thank you for noting this error. We have corrected this in Figure 1.**

Most of the 119 studies which formed the basis of this database have not been cited. I realise there are reference limits. But I do believe that readers should be able to easily see and subsequently find which studies have been a part of this study. I suggest having another supplementary file (table) that lists the full citation for each of the 119 studies.

**We have added a Supplementary Information file that includes the full references for all included studies, and we have cited this document in the text (line 244).**

Part III – Minor Issues: Editorial and Data Presentation Modifications

Reviewer #1: See above

Reviewer #2: (No Response)

Reviewer #3: Line by line comments:
Line 30: Change '25' to '30' years.

**We have made this change.**

Line 75: There is a more recent version of reference 3. I would recommend either of the following:
• Walker PJ, Siddell SG, Lefkowitz EJ, Mushegian AR, Adriaenssens EM, Alfenas-Zerbini P, Dempsey DM, Dutilh BE, García ML, Curtis Hendrickson R, Junglen S, Krupovic M, Kuhn JH, Lambert AJ, Łobocka M, Oksanen HM, Orton RJ, Robertson DL, Rubino L, Sabanadzovic S, Simmonds P, Smith DB, Suzuki N, Van Doorslaer K, Vandamme AM, Varsani A, Zerbini FM. Recent changes to virus taxonomy ratified by the International Committee on Taxonomy of Viruses (2022). Arch Virol. 2022 Aug 23. doi: 10.1007/s00705-022-05516-5.
• or direct citation of the web site "International Committee on Taxonomy of Viruses (ICTV): https://ictv.global/taxonomy/"

**Thank you for the suggestion; we have updated the citation to Walker et al. 2022.**

Line 115: please explain what 'PRISMA' is. For someone new to the area, they won't know what this is.

**We have added a definition of the acronym here (lines 116-117), and we later cite the PRISMA protocol in our Methods section (line 130).**

Line 152: Blood is listed as the most common sample type, representing 29% of all samples. Can you please additionally differentiate between serum used for serology and blood (RBCs) used for PCR and/or virus isolations.

**We have added another sentence with a further breakdown of detection methods used on the blood samples (lines 267-268):**

**“Of the blood samples, 90.9% were used for serological testing, 8.7% for PCR, and 0.5% for viral isolation.”**

Line 214: “16 of the 78 recognised species in the family Paramyxoviridae have been detected ….”. Can you please elaborate what you mean by ‘detected’? Is it PCR positive, or antibody positive, or virus isolation positive, or all of these?

**We have now clarified that we are referring to any detection method (line 330). In practice, this means initial detection by PCR or viral isolation, followed by subsequent, less-specific serological studies.**

Line 221: Rather than using the word ‘overstudied’, as this might send the wrong message and I am sure that the researchers in this field would not agree, can you please use the phrase ‘over-represented in the studies’. I agree with the use of the word ‘under studied’.

**We have made this change throughout (lines 326, 338).**

Line 348: change wording as follows - the isolation of Hendra and Nipah viruses from flying foxes in 1996 and 2000 respectively the early 2000s.

**We have made this change (lines 465-466).**

Line 512: In this section on antibody positivity, you need to consider sero-cross reactivity. It just needs to be mentioned that there is low specificity for most diagnostic tests and we see cross reactivity with closely related viruses.

**We have added a sentence clarifying this (lines 216-219):**

**“We note that seroprevalence is determined with different methods and cutoff values across the studies and that serological assays also capture cross-reactivity with different antigens; here, we seek to understand broad patterns in general paramyxovirus seroprevalence.”**

Figure S1: I think this is very informative and would recommend moving this figure to the main body of the paper (if this is possible).

**The manuscript already includes six figures, so we hesitate to include a methods figure in addition. However, we would be happy to move the figure into the body of the manuscript if the editor prefers this.**
