# Supplementary material for: Paramyxoviruses in Old World fruit bats (Pteropodidae): An open database and synthesis of sampling effort, viral positivity, and coevolution
Source: PLoS Negl Trop Dis. 2025 Nov 7;19(11):e0013698. doi: 10.1371/journal.pntd.0013698 (PMC12617870; doi:10.1371/journal.pntd.0013698)
Supplement: Attachment — Submitted filename: Juman et al. R2R.pdf [file pntd.0013698.s011.pdf]

October 24, 2025

Dear Dr. Schountz,

I have uploaded a revised version of our manuscript, “Paramyxoviruses in Old World fruit bats (Pteropodidae): an open database and synthesis of sampling effort, viral positivity, and coevolution” (PNTD-D-25-01418) into the Editorial Manager portal, and it should be ready for your inspection. We appreciated the opportunity to revise the manuscript as well as the constructive feedback from reviewers. In revising, we paid close attention to the reviewer comments. This led us to elaborate on several points in the discussion and include some additional plots and analyses in our supplementary file.

Below, we included all of the editor and reviewer comments, followed by our responses to them (in **bold**). Cited line numbers correspond to the revised manuscript with changes tracked.

Thank you again for your consideration. Please do not hesitate to contact me if you have any questions.

Sincerely,
Maya Juman

Editor Comments:

Dear Dr. Juman, the reviewers have identified several minor issues with the manuscript that should be addressed. Reviewer 2, in particular, has raised several points for clarification. Please respond to each of these points for the resubmission.

Thank you for the opportunity to address these issues.

Reviewer Comments:

Methods

Reviewer #1: Great

Reviewer #2: Minor Revision

1. Author should provide the total number of samples included for each testing method (PCR, ELISA, and virus isolation). To improve readability, include explicit numbers and positivity rates in the main text for each method and sample type (e.g., n=XX, YY% positive). (Line 255)

We have added the sample sizes to this paragraph (line 259 onward), as well as mean positivity for testing methods:

"Of the total records, 66% (*n* = 972; 3.0% mean positivity) involve PCR detection attempts, 27% (*n* = 393; 25.8% mean positivity) involve serological detection attempts, and 7% (*n* = 111; 0.7% mean positivity) involve viral isolation attempts… The most common sample types in our dataset are blood (29%; n = 425), urine (27%; n = 405), swabs (22%; n = 323), and tissue (16%; n = 232)."

A detailed breakdown of positivity rates stratified by sample type and testing method is provided in Supplementary Table 1.

2. You mention that PCR prevalence and seroprevalence were not significantly influenced by the year of collection, suggesting little change across decades. I recommend to provide a sensitivity analysis to test whether methodological differences across time might obscure temporal trends? (Line 546)

Specific detection methods (e.g., within PCR and within serology) could not be included in our model due to collinearity issues. We have now added stacked barplots to the supplement (Supplementary Fig. 2) showing that changes in diagnostic method track with time (a statistically significant association). This suggests that including sampling year as a predictor in our models accounts for these changes in diagnostic methods over time (and year was insignificant, suggesting that these changes have had little effect on prevalence or seroprevalence). We have now explained this further in our methods (lines 221-225) and discussion (551-553):

“Methodological variation within datasets could not be included in these models due to collinearity with other predictors; however, changes in diagnostic methods track with time (e.g., a shift from RT-PCR to metagenomic methods for PCR detection and a shift from neutralization assays to ELISA and multiplexed assays for serological detection), such that including sampling year as a predictor accounts for this variation (Supplementary Fig. 2)”

“Over time, there has been a shift in PCR detection methods from targeted RT-PCR to metagenomic approaches; in serological studies, neutralization assays have been supplanted by multiplexed Luminex assays and ELISA (Supplementary Fig. 2).”

Results

Reviewer #1: Yes - all good

Reviewer #2: Minor Revision

1. While pooled swab positivity is mentioned (5.4%) and detailed in supplementary data, the main text does not specify overall positivity or breakdown of sample types with positivity (e.g., oral or rectal swab). Add this information to the results for completeness, as it could inform non-invasive sampling strategies. (Lines 430–431)

We have added this information to the results (lines 441-449):

“We found that PCR prevalence was highest in spleen samples (6.9%), followed by pooled tissue samples from multiple organs (6.7%), pooled fecal samples from likely multiple individuals (6.5%), pooled swabs/samples (5.4%), and pooled urine samples from likely multiple individuals (4.2%) (Fig. 5). All other tissue or sample types yielded around or under 2% prevalence: reproductive organs (2.1%); urine samples from single individuals (1.7%); kidney (1.0%); oral swabs (0.8%); fecal, rectal, or anal swabs/samples (0.8%); blood or serum (0.5%); nasal swabs (0.4%); fecal samples from single individuals (0.2%); and liver (0.1%). Lung, intestine, and skin swabs all had 0% PCR prevalence.”

2. The meta-analysis synthesizes data from studies spanning 1971–2023, but study periods are not explicitly included as a factor. Please add this to the methods and results sections, as temporal clustering of studies could influence overall prevalence estimates due to varying environmental or methodological factors.

We include sampling year in our meta-analysis models, as well as a random effect of study ID to account for other potential sources of heterogeneity across studies. We have now computed the temporal autocorrelation function for both PCR prevalence and seroprevalence and added these plots to our supplement (Supplementary Fig. 3). Sharp declines in autocorrelation after the zero lag suggest limited temporal clustering of studies, so including continuous sampling year as a predictor effectively accounts for any variation across time (lines 226-227):

“Limited temporal autocorrelation in both PCR prevalence and seroprevalence also suggests that sampling year effectively controls for any temporal clustering of studies (Supplementary Fig. 3).”

Conclusions

Reviewer #1: Yes

Reviewer #2: Revision required as per recommendations

1. Your findings highlight that pararubulaviruses are understudied compared to henipaviruses. Could you elaborate on why this bias exists in details? (Line 511)

We have added a sentence elaborating on why this bias exists (lines 532-534):

“This skew is driven by the disproportionate focus on henipavirus detection after the discovery of Hendra and Nipah viruses in flying foxes in 1996 and 2000.”

2. Since PCR prevalence and seroprevalence represent different methodologies, so require more explanation to avoid potential confounding and better highlight method-specific patterns. Explain it in the results and discussion section.

We have added a sentence explaining this in the results (lines 408-411) and in the discussion (lines 589-591):

“These models were fitted to aggregated datasets of PCR prevalence and seroprevalence which include different methodological approaches, primers and gene targets, and other sources of variation in viral positivity.”

“Consequently, our models treat all PCR and all serological detection attempts as comparable to one another (though weighted by sample size), when in reality methodologies can differ widely across studies.”

We previously acknowledged this in the methods as well (lines 216-219):

“We note that seroprevalence is determined with different methods and cutoff values across the studies and that serological assays also capture cross-reactivity with different antigens; here, we seek to understand broad patterns in general paramyxovirus seroprevalence.”

3. The finding of higher prevalence in spleen samples (6.89%) is highlighted (Figure 5) may be affected by redundancy, since individuals positive in other tissues (e.g., swabs) could bias detection rates, especially given the small sample size. Could you clarify this point and discuss the limitations of sample size in tissue-specific conclusions?

We have now included this in our limitations paragraph (see point 4 below) on lines 596-599:

“Other limitations of this study include redundancy across samples from the same individual bat (e.g., the screening of tissue samples from bats already found to be positive, which artificially inflates positivity estimates) as well as reporting gaps in the literature, as our analysis only includes published data.”

4. I recommend adding a separate paragraph explicitly outlining the study’s limitations (e.g., heterogeneous methods across studies, reporting gaps, sample redundancy, uneven sample sizes, geographic under-sampling in Africa, reliance on published data only).

We have created a separate paragraph that discusses these limitations (starting on line 570):

“Syntheses and meta-analyses such as this can be limited in scope and statistical robustness by data gaps as well as a lack of stratified and standardized metadata reported in existing studies. Our study is inherently restricted by between-study variation at a finer level than what is captured in our dataset and in many of the original studies (e.g., sampling protocols, PCR primers, laboratory protocols, etc). Consequently, our models treat all PCR and all serological detection attempts as comparable to one another (though weighted by sample size), when in reality methodologies can differ widely across studies. Where possible, researchers should report the specific tissue types, localities, sampling dates, taxonomic identifications, and other metadata included in pooled samples; we also suggest providing detection estimates at as fine-grained a level as possible to enable more detailed analysis and between-study comparison of pteropodid–paramyxovirus associations. This will help inform future meta-analyses as well as sampling protocols for surveillance of wild bats and museum specimens. Other limitations of this study include redundancy across samples from the same individual bat (e.g., the screening of tissue samples from bats already found to be positive, which artificially inflates positivity estimates) as well as reporting gaps in the literature, as our analysis only includes published data.”

5. Virus isolation is notably low compare to PCR, likely due to requirements for BSL-4 facilities that is very limited worldwide, especially for henipaviruses. Expand on this in the discussion section to explain why PCR dominates and implications for confirming viable virus.

We have now elaborated on the potential reasons behind this methodological bias in the discussion (lines 565-569):

“However, only 7% of records involved viral isolation attempts, the gold standard for confirming viral presence in a sample. This is due in part to the strict biosafety requirements and resources (e.g., BSL-4 facilities) involved in viral isolation for henipaviruses, which inhibit detection attempts in countries with limited resources where these viruses are known to circulate.”

6. Pooled urine and fecal samples from roosts show higher positivity, possibly because they mix

contributions from multiple bats. Discuss this in the results and discussion section to guide interpretation for colony-based surveillance.

We have clarified that multiple individuals can contribute to pooled samples in the results (lines 441-444) and have added a sentence to the discussion (lines 561-563) explicitly mentioning this:

"We found that PCR prevalence was highest in spleen samples (6.9%), followed by pooled tissue samples from multiple organs (6.7%), pooled fecal samples from likely multiple individuals (6.5%), pooled swabs/samples (5.4%), and pooled urine samples from likely multiple individuals (4.2%)."

"Pooled samples generally show higher positivity, as they contain samples from likely multiple individuals, raising the likelihood of a positive result."

<u>Editorial and Data Presentation Modifications?</u>

Reviewer #1: Reference 3 does need to be changed to Walker et al (as is indicated in the author's response).

We appreciate the reminder. This was an oversight and has been corrected (lines 623-625).

Reviewer #2: Accept

<u>Summary and General Comments</u>

Reviewer #1: The authors have clearly addressed all 3 reviewer's comments and suggestions. There was only one minor change that was not done - and that was updating reference 3 to Walker et al.

This has been corrected (lines 623-625).

I also want to commend the authors for their considered responses to Reviewer 1 who was unprofessional and dismissive in their approach to providing constructive feedback.

Reviewer #2: The paper is clearly written, conceptually sound, and well-organized. The results are important for understanding the ecology of paramyxoviruses in pteropodid bats and for directing future monitoring activities. The public repository (GitHub) is an important resource for the scientific community. However, the manuscript has a few points for improvement to enhance clarity.

We thank Reviewer 2 for the detailed feedback and have addressed all points listed above.
